# Peptide Inhibitors of Dengue-Virus Entry Target a Late-Stage Fusion Intermediate

**DOI:** 10.1371/journal.ppat.1000851

**Published:** 2010-04-08

**Authors:** Aaron G. Schmidt, Priscilla L. Yang, Stephen C. Harrison

**Affiliations:** 1 Jack and Eileen Connors Laboratory of Structural Biology, Department of Biological Chemistry and Molecular Pharmacology, Harvard Medical School, Boston, Massachusetts, United States of America; 2 Department of Microbiology and Molecular Genetics, Harvard Medical School, Boston, Massachusetts, United States of America; 3 Howard Hughes Medical Institute, Harvard Medical School, Boston, Massachusetts, United States of America; Washington University School of Medicine, United States of America

## Abstract

The mechanism of membrane fusion by “class II” viral fusion proteins follows a pathway that involves large-scale domain rearrangements of the envelope glycoprotein (E) and a transition from dimers to trimers. The rearrangement is believed to proceed by an outward rotation of the E ectodomain after loss of the dimer interface, followed by a reassociation into extended trimers. The ∼55-aa-residue, membrane proximal “stem” can then zip up along domain II, bringing together the transmembrane segments of the C-terminus and the fusion loops at the tip of domain II. We find that peptides derived from the stem of dengue-virus E bind stem-less E trimer, which models a conformational intermediate. *In vitro* assays demonstrate that these peptides specifically block viral fusion. The peptides inhibit infectivity with potency proportional to their affinity for the conformational intermediate, even when free peptide is removed from a preincubated inoculum before infecting cells. We conclude that peptides bind virions before attachment and are carried with virions into endosomes, the compartment in which acidification initiates fusion. Binding depends on particle dynamics, as there is no inhibition of infectivity if preincubation and separation are at 4°C rather than 37°C. We propose a two-step model for the mechanism of fusion inhibition. Targeting a viral entry pathway can be an effective way to block infection. Our data, which support and extend proposed mechanisms for how the E conformational change promotes membrane fusion, suggest strategies for inhibiting flavivirus entry.

## Author Summary

Enveloped viruses must overcome a succession of cellular barriers before establishing infection. One obstacle is fusion of viral and cellular membranes. Rearrangements of proteins on the viral surface facilitate fusion and subsequent delivery of the viral genome into the cytosol. In this study, we probed the fusion-promoting rearrangement of the dengue-virus envelope (E) protein. Peptides derived from the membrane proximal “stem” of E bind to a form of recombinant E that represents a late-stage intermediate in its low-pH triggered conformational change. The binding mimics a key step in the fusion-promoting process. We find that these stem peptides also inhibit viral infectivity, with potency proportional to their affinity for E, and that they do so by specifically blocking fusion. We provide evidence that inhibition is a two-step process: an initial, nonspecific interaction of the peptide with the viral membrane, followed by specific binding to E, as the protein undergoes conformational rearrangement. The initial step explains how the virus can carry the peptide into an endosome—a necessary step, because the binding surface on E becomes available only after exposure to low pH. This work extends the model of flavivirus fusion, and suggests strategies for targeting viruses that penetrate from endosomes.

## Introduction

Membrane fusion is a critical step for infectious entry of enveloped viruses into cells [1]. A viral “fusion protein” facilitates this process, generally in response to molecular cues specific for the cellular compartment in which viral penetration occurs. For example, dengue and other flaviviruses penetrate from endosomes, following uptake by clathrin-mediated endocytosis [2],[3], and proton binding is the immediate fusion trigger [4].

**Figure 1:**
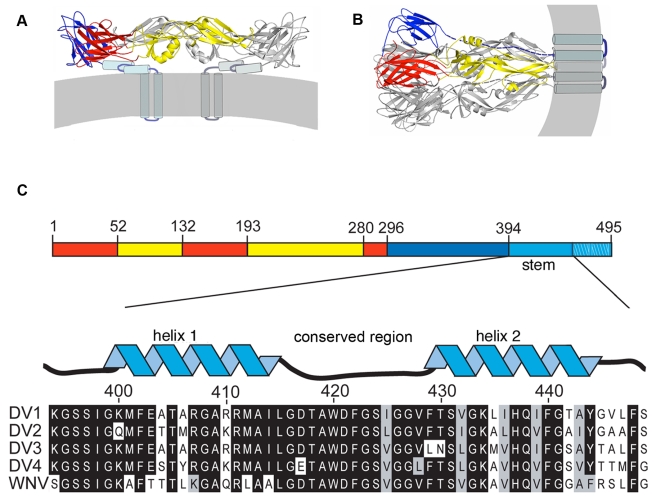
Conformational states of the dengue virus E protein and sequences of the membrane-proximal “stem”. (A) Structure of E in the dimeric conformation present on the virion surface prior to low-pH exposure. The view is tangential to the viral membrane (gray stripe). The sE component (residues 1–395) of the ectodomain is in ribbon representation, with domains I, II, and III in red, yellow, and blue, respectively. The stem (residues 396–447) is shown as a helix-loop-helix, modeled from a cryoEM reconstruction [7]. The transmembrane anchor is an α-helical hairpin. (B) E in the trimeric conformation it adopts following a low-pH induced conformational change. Dashed lines indicate the likely position of the stem segments. (C) Linear representation of the E polypeptide chain, illustrating the regions that fold into the structures shown in A and B. The stem is shown “magnified”, together with stem sequences from dengue serotypes 1–4 and from West Nile virus (WNV). The designations of “helix 1”, “conserved region”, and “helix 2” follow.

The flaviviruses are insect-borne agents with positive-strand RNA genomes packaged into compact particles, about 500 Å in diameter [5]. Their fusion protein, known as E, is the principal external protein of the virion. It is made as part of a polyprotein, which includes a “chaperone” protein, designated prM (precursor of M). Cleavage of prM during viral maturation releases most of its ectodomain and promotes formation of a well-ordered lattice of 90 E dimers on the virion surface [6],[7]. When the pH drops below about 6.2, E undergoes a large-scale conformational rearrangement that includes dissociation of the dimer and reconfiguration of the subunits into trimers (Fig. 1A,B) [8]. At an intermediate stage in this complex molecular reorganization, a hydrophobic “fusion loop” at one end of the extended E subunit inserts into the outer leaflet of the target membrane bilayer [9],[10],[11],[12]. Further rearrangement then draws together the fusion loop and the transmembrane segment anchoring E in the viral membrane. The latter step forces the two membranes together, allowing fusion to ensue.

This molecular description of flavivirus fusion derives in part from crystal structures of several flavivirus E proteins in conformations corresponding to prefusion dimers and to postfusion trimers (Fig. 1A) [9],[11],[12],[13],[14]. These structure determinations were made possible by C-terminal truncation of the E ectodomain, through proteolytic treatment or recombinant expression, to increase protein solubility and biochemical stability [15]. These soluble forms of E (“sE”) include only the first 395 of the approximately 445 ectodomain residues; they lack a membrane-proximal region called the “stem” (Fig. 1C) that is substantially conserved among flaviviruses. Intermediate-resolution cryoEM reconstructions show that in the prefusion dimer, the stem segment folds into a helix-loop-helix sandwiched between the rest of the ectodomain and the membrane [7],[16]. In the postfusion conformation, it has been inferred that the stem extends along the edge of domain II, which bears the fusion loop at its tip (Fig. 1) [11].

In the postfusion conformation of E, the fusion loops and the C-terminal transmembrane anchors of all three subunits cluster at one end of the rearranged protein (Fig. 1B). The driving force for pinching together of the two membranes appears to come from contacts made by domain III, as it folds back against domain I at the base of the hairpin, and by the stem with domain II, as it “zips” up along domain II. A consequence of this picture is that interfering with either of these interfaces – for example, by a soluble form of domain III or of the stem – will probably prevent viral fusion. A well-known precedent is T-20/entfuvirtide, a peptide inhibitor of HIV-1 infectivity [17],[18],[19],[20]. T-20 is a soluble fragment of gp41 that blocks the analogous zipping-up step in gp41 refolding. Kielian and co-workers have shown that soluble dengue E-protein domain III can inhibit entry of dengue virus bound at the cell surface when fusion is triggered by transient acidification, and they have made similar observations for the related alphavirus fusion protein, E1 [21]. The likely mechanism involves binding of the soluble domain to the extended intermediate structure, thereby aborting the internal fold-back. One report also suggests that a stem peptide can inhibit dengue virus infection in a conventional infectivity assay, but with no mechanistic analysis [22]. The observation is puzzling, because we might expect a site for specific stem-peptide binding to be present only after induction of a conformational transition by the low pH of an endosome and an externally applied peptide to be inactive unless carried into the endosome by some non-specific process.

In the work reported here, we show that peptides derived from the dengue E protein stem indeed bind the trimeric, postfusion sE conformer, but not the prefusion dimer. The sequence specificity of this interaction is consistent with our prediction, based on the trimeric sE structure, that such peptides should be able to dock against domain II. We also show that the same stem peptides can inhibit viral infectivity by binding the virion at neutral pH before cell attachment and, presumably, by accompanying the virus particles into endosomes. Hydrophobic interactions between the C-terminal segment of the peptide and the viral membrane contribute to virion binding and hence to inhibiting infection; sequence-specific contacts may have a role in initial binding, in addition to their principal inhibitory contribution when internalized peptide binds rearranging E protein, after the virus has encountered a lowered pH.

## Results

### Binding of stem peptides with sE

The order of conformational changes in E outlined in the Introduction suggests that stem peptides might bind the trimer-clustered domain-II regions of sE. That is, because the entire stem is missing from the sE fragment, the sE trimer in effect represents an intermediate state in the conformational transition, just before the stem zips up along domain II. Fig. 1C shows the stem sequences from DV serotypes as well as the closely related West Nile Virus (WNV) and illustrates their nearly complete conservation. We made a set of synthetic peptides, all based on the DV2 consensus sequence, for affinity measurements. The peptides were conjugated to FITC at their N-termini, and their affinities for prefusion sE dimer and postfusion sE trimer were measured with a fluorescence polarization assay.

We produced the trimeric postfusion form of sE following the protocol of Modis et al (2004), which derived in turn from earlier work [23] on tick-borne encephalitis (TBE) virus. Acidification of sE dimer in the presence of liposomes leads to dimer dissociation, insertion of the fusion loop into the outer liposome leaflet, and catalysis of trimer formation by the enforced alignment of monomers on the membrane surface. The sE remains trimeric after neutralization; it is soluble in the presence of non-ionic detergents.

**Figure 2:**
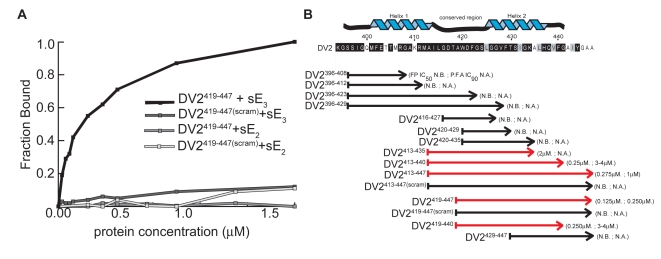
Binding and affinities of stem-derived peptides with DV2 sE(1–395) and inhibition of DV2 infectivity. (A) Binding of a representative stem-peptide, DV2^419–447^, and of a peptide with the same composition but a scrambled amino-acid sequence, determined by fluorescence anisotropy. The fraction bound is plotted as a function of sE protein concentration (in µM). sE_2_ and sE_3_ refer to dimer and trimer, respectively. Trimer-binding curves were corrected for low levels of binding to the UDM present in the sE_3_ preparations. (B) The dissociation constants for various stem peptides were estimated by the concentration of half-maximal change in fluorescence polarization (FP IC50), as described in Methods. The effect on viral infectivity is shown as “IC90”, the concentration of peptide that reduced viral yield (of DV2 on BHK cells) to 10% of the control. N.B., no binding; N.A., no activity. Each FP IC50 is an average of at least three independent experiments, each done in triplicate. Each IC90 is from an assay set up in duplicate and plaqued in triplicate.

We measured binding isotherms by adding increasing amounts of either the prefusion or the postfusion conformer of sE to a constant concentration of FITC-tagged peptide. Dissociation constants (K_d_) were calculated as described in Methods. Peptide DV2^419–447^ binds selectively to the postfusion conformer of sE; a scrambled version of DV2^419–447^ (DV2^419–447(scram)^), used as a specificity control, binds neither conformer (Fig. 2A). Results from this series of experiments are summarized in Figure 2B. Peptides containing the C-terminal half of the dengue virus type 2 stem bind the sE postfusion trimer, while those derived exclusively from the N-terminal half do not. Peptide 419–447 (DV2^419–447^) has the lowest K_d_ (∼150 nM), and we have chosen to use it for many of the subsequent studies reported below.

We confirmed the specificity of stem peptides for the postfusion sE conformer by showing that a biotinylated derivative of DV2^419–447^ stably associates with trimeric sE, using the “pull-down” protocol described in Methods. As shown in Fig. S1, biotinyl-DV2^419–447^ associates tightly with sE trimer but not with sE dimer. As a control for non-specific interaction of the biotinylated peptide with a detergent-solubilized protein, we used an irrelevant membrane protein (the mitochondrial uncoupling protein, UCP) and found no evidence for any association (data not shown).

### Stem peptides inhibit viral fusion

If peptide binding to sE trimer mimics an association of stem with domain II during the fusion-promoting conformational change, then the bound peptides should block the final “zipping-up” step and thereby prevent fusion. We used two methods to monitor directly the effects of stem-derived peptides on dengue-virus fusion with liposomes. One is a trypsin susceptibility assay, which detects exposure of the viral core protein to liposome-encapsulated protease as a measure of complete membrane fusion and content mixing. The other is a pyrene fluorescence assay that has been validated in many published studies of alphaviruses and flaviviruses as a sensitive measure of hemifusion, an obligatory intermediate step in the merging of two bilayers [24],[25],[26].

**Figure 3:**
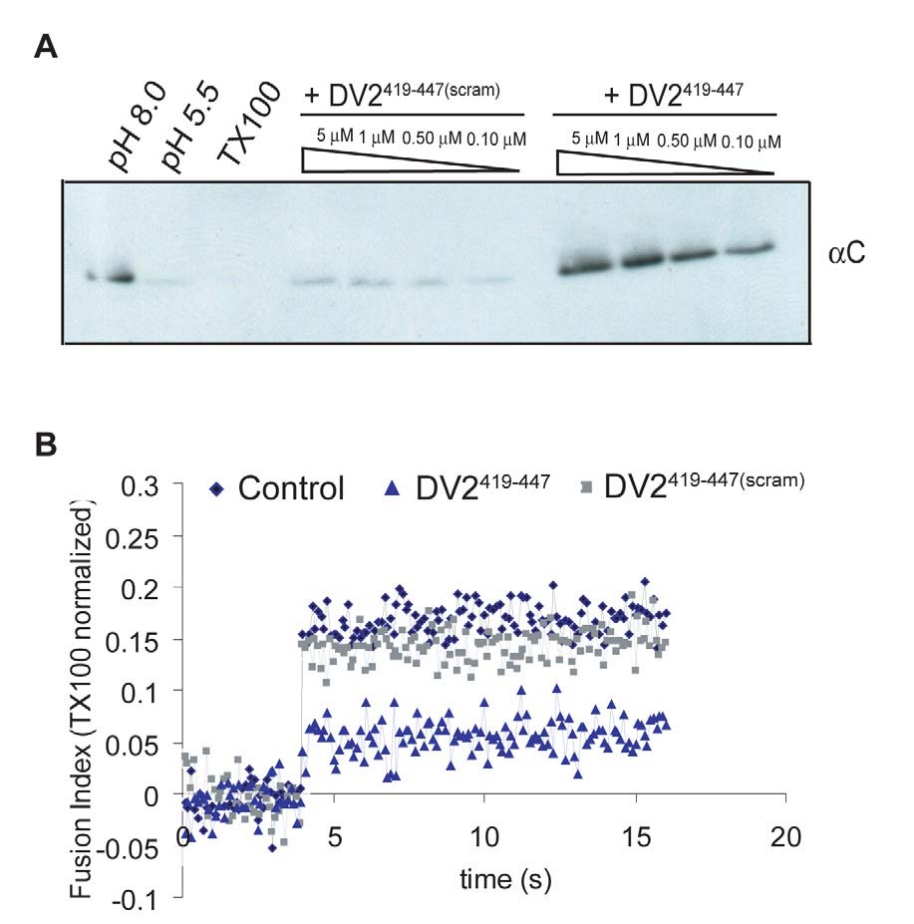
Inhibition of fusion of virus and liposomes. (A) Effect on fusion-pore formation (content mixing) of preincubating virions with DV2^419–447^ or DV2^419–447(scram)^. Virions and peptides were incubated with liposomes encapsulating trypsin and acidified to pH = 5.5. Following back-neutralization and incubation for 1hr at 37C, samples were prepared for SDS-PAGE by TCA precipitation and immunoblotted with anti-C antibody. Fusion leads to exposure of capsid protein to trypsin and loss of the corresponding band. (B) Effect on the hemifusion step of preincubating pyrene-labeled virions with DV2^419–447^ or DV2^419–447(scram)^. Hemifusion was measured by the decrease in pyrene excimer intensity when virions were acidified to pH = 5.5 in the presence of liposomes. Loss of excimer fluorescence following dissolution of the viral membrane by TX100 was taken as 100%. Data represent three independent experiments.

For the content-mixing assay, liposomes encapsulating trypsin were made by extrusion in the presence of the protease. Unincorporated trypsin was removed by size exclusion chromatography. Virions and trypsin-containing liposomes were incubated at 37°C at neutral and low pH, in the presence of DV2^419–447^ or DV2^419–447(scram)^; reactions were back-neutralized and incubated for 1hr at 37°C. Fusion, triggered by exposure to low pH, will deposit the viral core into the liposome interior, exposing the capsid protein to protease. The results in Figure 3A show that in the presence of DV2^419–447^, there is no more capsid degradation than in the pH 8.0 control, whereas DV2^419–447(scram)^ has no protective effect with respect to the pH 5.5 treated reaction. Complete lysis of the liposomes with TX100 demonstrates that there is sufficient trypsin encapsulated to digest the capsid protein completely. DV2^419–447^ inhibits membrane fusion in this model system at a step prior to content mixing.

For the pyrene fluorescence measurements, virus was grown in medium supplemented with pyrene-hexadecanoic acid, which incorporates into membrane lipids. The pyrene labels the tip of the fatty-acid chain near the bilayer midplane; an excited fluorophore in one leaflet can collide with an unexcited fluorophore in the other leaflet, generating an excimer with an altered emission spectrum [27]. Fusion with an unlabeled liposome dilutes the pyrene labeled lipids at the hemifusion stage, leading to a decrease in fluorescence at the wavelength of the excimer emission maximum. The data in Fig. 3B show that addition of 1µM DV2^419–447^ substantially reduced the extent of hemifusion with respect to the effects of DV2^419–447(scram)^ and DMSO as controls.

### Effect of stem peptides on viral infectivity

To examine the effect of the stem peptides on dengue virus infectivity, we infected cells in the presence of varying peptide concentrations and then measured viral yield 24 hours post-infection by a standard viral plaque assay (see Methods). Stock solutions of peptides were made in DMSO, and the final DMSO concentration was always less than 1% when the stock was diluted into medium. Peptides derived from the C-terminal part of the stem inhibit viral replication. As shown in Fig. 2, the extent of inhibition correlates strongly with the affinity of the peptide for the sE trimer, as determined by fluorescence polarization measurements. Peptides that include the entire “helix II” segment are particularly active. DV2^413–447^ and DV2^419–447^ are the strongest inhibitors; neither scrambled version, DV2^413–447(scram)^ or DV2^419–447(scram)^, has any effect on viral titre.

### Effect of order of addition

At what stage in the infectious cycle do stem peptides interfere with viral replication? The results shown in Fig. 2 were obtained from experiments in which peptide and virus were preincubated before application of the inoculum to cells. Thus, the peptides could associate with virions prior to adsorption of the latter to cells and inhibit completion of the envelope protein conformational transition after virion uptake into endosomes. Alternatively, it is possible that the peptides concentrate in endosomes and associate with virions only in a low-pH environment. Either explanation appears to present a puzzle. At the 500 nM concentration sufficient, in the case of the most effective peptides, to inhibit viral yields, an early endosome of 1500 Å diameter would be expected to contain only 1–2 peptides in the absence of some mechanism to concentrate the peptide. This number is probably too small to inhibit viral entry, as estimates for the number of antibodies required to neutralize dengue virus range from 20 upwards, and it is likely that at least the same number of peptides would be needed [28],[29]. Yet, the inhibitory activity of the peptides correlates with their binding to sE trimer, and the trimer transition should occur only after endosomal acidification. The correlation also appears to rule out a third possibility – that the peptides stimulate or inhibit a cell-signaling pathway, rather than inhibiting infection through direct interaction with virions.

**Figure 4:**
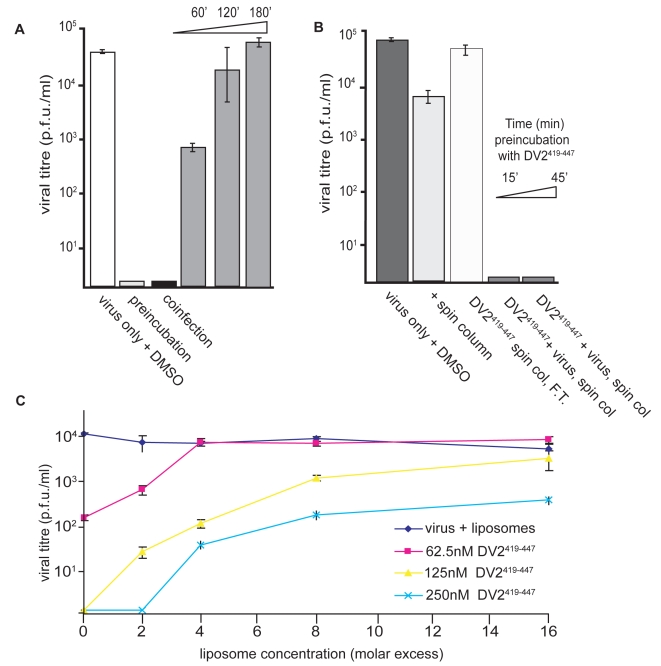
Inhibition and reversibility of viral infectivity by DV2^419–447^. (A) Influence of the time of peptide addition on viral yield. DV2^419–447^ (or DMSO carrier, for the control) was added to virus at 37°C, 45 minutes (preincubation) or immediately (coinfection) before applying the inoculum to cells, or at various time points postinfection (as shown by the time ramp). For the 0′ time point, peptide was added promptly after adding the inoculum. (B) Effect of separating peptide and virus prior to infection. DV2^419–447^ (10 µM) was preincubated with virus for 15′ or 45′ at 37*C, and unbound peptide was removed by rapid passage of the inoculum through a size-exclusion spin-column prior to addition to cells. Passage of virus (plus DMSO carrier) through the column reduced titre by about 10-fold. The flow-through fraction (F.T.) of medium plus peptide had no inhibitory effect on virus added subsequently, consistent with retention of all unbound peptide by the matrix. (C) Reversibility of stem peptide inhibition. Viral inoculum was preincubated with DV2^419–447^ for 10′ at 37C. Liposomes were then added to the peptide∶inoculum, in the molar excess of lipid molecules to peptides shown and incubated for an additional 45′. The inoculum was added to cells and harvested 24hrs later. An inoculum preincubated with liposomes alone has no loss in viral titre.

To resolve these apparent contradictions, we carried out a series of order-of-addition experiments using either DV2^419–447^ or DV2^419–447(sol)^. The latter spans the same sequence (419–447), but has an additional “solubility tag”, of sequence RGKGR, at its C-terminus. The hydrophobicity of the stem peptide causes it to aggregate almost immediately upon dilution from DMSO into the medium, decreasing its effective concentration as the incubation proceeds (data not shown); the solubility tag reduces aggregation and stabilizes the peptide concentration. Pre-incubation of BHK-21 monolayers with 10 µM DV2^419–447^ for various times, followed by infection with DV2 at a multiplicity of infection (MOI) 1 after washing the cells free of the peptide, did not affect the viral yield (data not shown). Thus, the peptide has no lasting effect on the capacity of the cells to support viral replication. Preincubation of virus with peptide DV2^419–447^ for various times prior to application to the monolayer leads to the same inhibition of infectivity observed when the two are mixed and applied immediately (Fig. 4A). Adding peptide at the end of the 1 hr interval used for the infection had a small inhibitory effect, suggesting the presence of some adsorbed but not yet endocytosed particles, whereas adding peptide 2 hr after application of the virus had no effect at all. We conclude that inhibition of infectivity results from a direct interaction of the peptide with virions and that the virion-bound peptide blocks an early step in infection.

In the experiments of Fig. 4, excess peptide was present during the incubation of virus and cells. Rapid passage of the virus through a short size-exclusion matrix, to remove excess peptide prior to application of the inoculum to the monolayer, did not reduce the extent of inhibition relative to a control passed similarly through the matrix but with no added peptide (Fig. 4B). We verified that the spin columns used for this treatment were effective in removing unbound peptide by showing that no inhibitory activity was present in the flow-through fraction after applying a 10 µM peptide solution (but no virus) to the column. Thus, peptide appears either to associate tightly with virions or to induce an irreversible, inactivating effect. Experiments described below rule out the latter interpretation.

### Reversibility of stem peptide inhibition

The experimental results in Fig. 4C show that inhibition of infectivity by stem peptides is reversible. We preincubated a viral inoculum with DV^419–447^ at 37°C, then added liposomes in varying molar excess. We could restore infectivity to levels approaching the control by adding a suitable excess of liposomes, into which the hydrophobic peptide could partition. The reversibility of inhibition by stem peptides suggests that non-specific membrane interactions may dominate the initial association of peptide and virion and that this sequestration may concentrate the peptide in the viral membrane for rapid association during the short lifetime of the E-protein fusion intermediate.

### Effect of temperature

**Figure 5:**
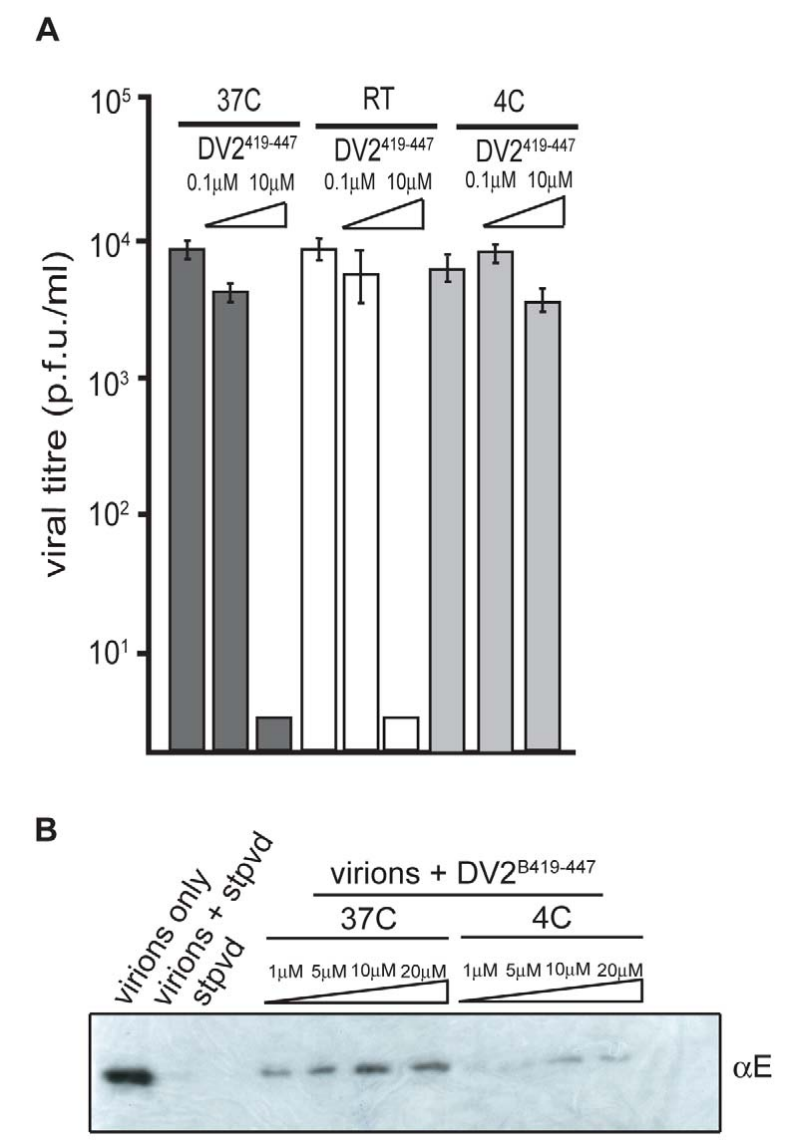
Temperature dependence of peptide-virus interaction. (A) Inhibition of infectivity after incubating virus with DV2^419–447^ at various temperatures, as indicated. RT, room temperature. (B) Association of biotinyl-DV2^419–447^ (DV2^B419–447^) with purified dengue virions at 4°C and 37°C (see Methods).

Together with the observation that stem peptides bind sE in its rearranged, trimeric conformation, but not the sE dimer, the data in the preceding section suggest that the peptide gains access to sites, either on domain II or on the viral membrane, that are occluded in the equilibrium structure deduced by fitting the dimer model into cryoEM reconstructions [7]. Indeed, when we carried out the peptide-virion incubations at 4°C rather than 37°C, followed by quick warming and immediate application to the BHK-21 cell monolayer, we found little or no effect on viral titre (Fig. 5A). The results suggest that dynamics of the virion surface permit access to transiently exposed sites on E, on the membrane, or on both.

### Direct detection of peptide association with virions

We examined the interaction of stem peptides and virions by incubating biotinyl-DV2^419–447^ with virions at 37°C for 45 minutes and then adding streptavidin resin (blocked with 10% fetal bovine serum to minimize non-specific association) to bind the peptide and any associated virus, which we detected by immunoblotting as shown in Fig. 5B. Binding of virions was efficient at pH 8, well above the pH threshold for fusion. As the concentration of peptide increased, we could detect increasing amounts of virus in the streptavidin eluate. Incubation of the virus with biotinyl-peptide at 4°C resulted in a noticeably lower yield of retained virions, consistent with the neutralization experiments (Fig. 5B).

**Figure 6:**
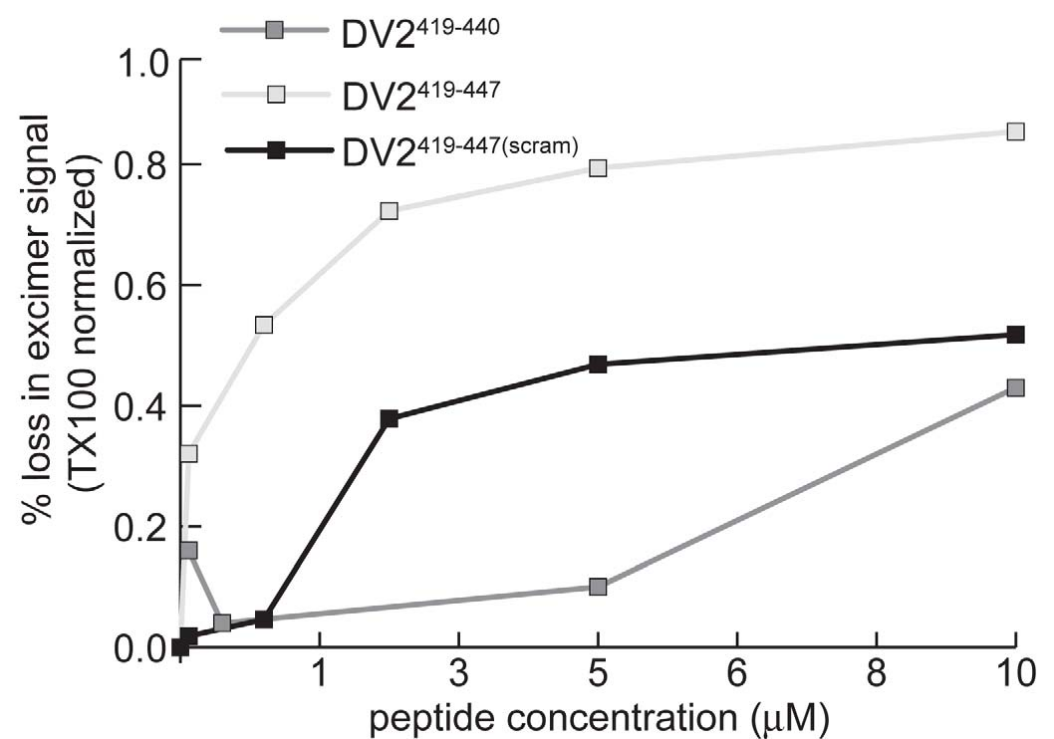
Stem peptides interact with the dengue virus membrane. Decrease in pyrene-excimer fluorescence intensity, as a function of the concentration of stem peptide added to pyrene-labeled virions, normalized to its complete loss upon addition of TX100.

To confirm association of stem peptides with virions we used the fluorescence properties of pyrene labeled virions as a direct measure of membrane interaction. We observed that addition of DV2^419–447^ produces a concentration dependent loss in excimer intensity (Fig. 6). We interpret this effect as a measure of interaction of the peptide with the bilayer. Membrane binding of stem peptides will decrease lipid mobility and hence decrease the likelihood of excimer formation. Consistent with this interpretation is the observation that DV2^419–447(scram)^, which is as hydrophobic as the unscrambled peptide, also reduces the excimer signal (Fig. 6). The loss of any regular pattern of amphipathicity in the scrambled peptide may account for the higher concentration required to achieve the same effect on the pyrene excimer as with wild-type peptide.

At the respective concentrations at which WT and scrambled peptides decrease the excimer intensity by 20%, the former decreases viral titre by 90%, while the latter has no effect. This comparison shows that loss of excimer signal is not due to a generalized membrane disruption that would inactivate the virus. (Even at saturating concentrations, the peptides do not eliminate excimer fluorescence completely, which we can do by adding TritonX-100.) The comparison also shows that association of a peptide with the membrane, as monitored by loss of excimer signal, is not sufficient for neutralization of infectivity. Membrane association appears to be necessary, however, as indicated by comparison of peptides DV2^419–447^ and DV2^419–440^ (Fig. 2). The latter lacks the hydrophobic segment between residues 441 and 447, but it binds soluble E trimer as tightly as does the former (Fig. 2). Only at concentrations at which the shorter peptide reduces the pyrene excimer intensity does it also neutralize (Fig. 2A and B). These results also suggest that in the conformation E reaches when fusion is complete, amino-acid residues in its stem may bind along domain II only about as far as 440. On a mature virion, residues between 441 and the transmembrane anchor probably interact with the viral membrane; following fusion, they may interact with the fused bilayers.

## Discussion

We have found that peptides derived from the membrane proximal region of the dengue virus E protein bind specifically to the postfusion conformer of sE, which is a model for a fusion intermediate. These peptides also inhibit viral infectivity – an initially surprising observation because transition of E to its postfusion conformation occurs in the sequestered environment of an endosome. We have shown that the inhibition results from direct interaction of the peptides with virions at neutral pH and hence that the virus carries the peptide with it into the cell. The peptides do not associate with virions at 4°C, suggesting that binding depends on dynamic properties of the viral envelope. We have further shown, using a liposome fusion assay, that the stem-derived peptides inhibit viral fusion at a step prior to hemifusion and fusion-pore formation.

How do these stem peptides inhibit fusion if their specific binding sites are not exposed until acidification in an endosome? One possible model is that the peptides bind to transiently exposed sites as the E protein fluctuates among an ensemble of conformations. A similar explanation has been offered to explain neutralization of dengue virus by antibodies for which the epitopes are blocked (or partly blocked) on the virion as modeled from the cryoEM structure [30]. The antibodies can trap a small fluctuation of the protein from its equilibrium conformation. The absence of peptide binding to dengue virions at low temperature is consistent with this type of model. Nonetheless, it seems unlikely that E could spontaneously fluctuate at neutral pH toward a conformation close to that of the postfusion trimer (the excursion of the domains and the rearrangement of the E protein are too extensive), and to consider a fluctuation-binding model, it is probably necessary to assume that the peptide associates with a single domain II, rather than with two that are clustered with each other as in the trimer. A small fluctuation could readily make an edge of domain II accessible to peptide: we know domain II to be hinged with respect to domain I and to adopt somewhat different hinge angles in different crystal forms of the sE dimer, suggesting a potential source of conformational heterogeneity [31]. Residual, uncleaved prM, which is present to varying degrees in most dengue inocula, is also likely to cause perturbations in the packing of E dimers on the virion surface [6],[32]. The association of prM with E tilts the latter outward from the particle surface, and we would expect that a small number of prM-E heterodimers would introduce dislocations and local disorder into the otherwise tightly packed lattice of E dimers covering the virion.

**Figure 7:**
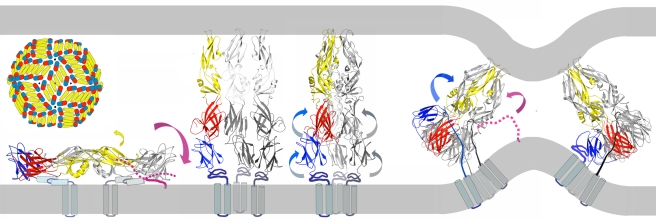
Model for inhibition of dengue-virus fusion by stem-derived peptides. The stem peptide (magenta-dotted line) may bind in an initial, protein-specific interaction to the edge of E, domain II, dynamically exposed at neutral pH. The yellow arrow shows a likely fluctuation in the orientation of domain II, based on observed variation in the domain I-domain II hinge angle. Alternatively, a two-step model may obtain, in which the stem peptide has an initial non-specific membrane interaction that anchors it through its C-terminus in the viral membrane (magenta-solid line) at neutral pH. As fusion within an endosome proceeds toward the final “zipping up” step, with collapse of the extended intermediate, the peptide, carried into the endosome by association with the viral membrane, could then make a high-affinity, protein-specific interaction with trimer-clustered domain II, blocking hemifusion and pore formation by preventing close approach of the fusion peptide and transmembrane anchor (later images of panel).

The results of our membrane-fusion assays with pyrene-labeled virus particles suggest an alternative, two-step model: step 1 is an initial hydrophobic association of peptide and viral membrane, and step 2 is a specific association with E during the low-pH triggered conformational transition (Fig. 7). Even a modest fluctuation of the closely packed E protein on the viral surface would give an exogenous peptide access to the underlying membrane bilayer, of which about two-thirds is free of intimate protein contacts [7]. A peptide that could associate with the membrane, e.g., as an amphipathic helix, would be carried with the virus particle into an endosome and would be available to “spring into place” upon transient exposure of its site on domain II, thereby blocking the fusion-promoting zipping-up of the E-protein stem. Only peptides with the appropriate sequence would be effective, consistent with the properties of the DV2^419–447(scram)^ peptide, which binds well to virions, but not to E trimer, and has no effect on viral infectivity. The two-step model is also consistent with the sequence dependence of peptide binding as measured by the effect of peptides on the intensity of the pyrene excimer in Fig. 6 and with the requirements for trimer binding in vitro as detected by fluorescence polarization (Fig. 2).

The last seven residues of DV2^419–447^ appear to be important for inhibitory activity. This membrane-proximal segment is the most uniformly hydrophobic sequence in the stem. Residues 441–447 contribute substantially to the neutralizing potency of the peptide (compare peptides in Fig. 2) but not to its affinity for the sE trimer. Moreover, reducing their hydrophobicity by substituting the corresponding residues from WNV greatly reduces inhibition of infectivity without affecting trimer binding (data not shown). Thus, these residues might be particularly important contributors to step 1 of the two-step model outlined above. If so, some specific peptide-protein contacts might occur in concert with peptide-lipid association. That is, residues 441–447, which do not contribute to E-protein binding, could associate with the membrane, while some part of the rest of the peptide made contact with its site on E, exposed by fluctuation or disorder as suggested in the single-step model. According to this combination of the two models above, non-specific membrane interactions and specific domain-II interactions would jointly recruit the peptides, rather than functioning only sequentially. Additional specific interactions could then form upon full exposure of domain II during the low-pH induced conformational transition.

Our data suggest strategies, which might otherwise have seemed unlikely, for inhibiting entry of viruses that fuse in endosomes or other internal compartments. Peptides from the membrane-proximal region of the HIV gp41 ectodomain are effective inhibitors of HIV entry, but fusion at the cell surface has made the transient intermediate in envelope refolding seem a more plausible target for HIV than for viruses with envelopes that expose an extended intermediate only after internalization [19],[20]. If peptides or small molecules can be concentrated in a viral membrane by tethering to a hydrophobic “tail”, then it may be possible to enhance the potency of inhibitors identified by a simple binding assay such as the one we have used here.

## Materials and Methods

### Peptide synthesis

Peptides were synthesized with standard Fmoc chemistry on ABI 431 Peptide Synthesizers at the Tufts University Core Facility (Boston, MA). They were purified using reverse phase HPLC and analyzed by mass spectrometry. Biotin and fluorescein-isothiocyanate (FITC) were conjugated to the N-terminus of the peptides via a beta-alanine linker.

### Liposomes

Liposomes [1-palmitoyl-2-oleoyl-sn-glycero-3-phosphocholine (POPC): 1-palmitoyl-2-oleoyl-sn-glycero-3-phosphoethanolamine (POPE) (Avanti Polar Lipids) and cholesterol (Sigma-Aldrich) at 1∶1∶2 molar ratio in TAN buffer (20mM triethanolamine, 100mM NaCl, pH 8.0)] were prepared by freeze-thaw extrusion through a 0.2 µ filter as described previously [11]. Liposomes were used within 2 days of preparation.

### Preparation of sE trimers

The postfusion sE trimer was produced as described [11]. Briefly, purified sE (Hawai'i Biotech) was incubated in the presence of liposomes for 15 mins at 37C, acidified with MES or sodium acetate to a final pH of 5.5, incubated for an additional 30 mins at 37C and at then at room temperature 1hr. Liposomes were solubilized with n-octyl-β-D-glucoside (β-OG) and n-undecyl-maltopyranoside (UDM). The solution was applied to a MonoS column (GE Healthcare) in 25mM sodium citrate pH 5.2, 100mM NaCl, 4mM UDM. sE trimer was eluted with a 1–1.5M NaCl step gradient and further purified by size-exclusion chromatography on Superdex 200 (GE Healthcare) in 10mM TEA, 80mM NaCl and 0.7mM UDM. Protein was dialyzed extensively using a 50-kDa molecular-weight cutoff membrane (Spectrapor).

### Fluorescence polarization

Binding experiments were carried out in Corning, low-volume 384 well microplates and analyzed in a *PerkinElmer EnVisions* instrument (excitation wavelength, 485nm; emission wavelength, 535 nm). Stock concentrations of FITC-conjugated peptide were made in DMSO and dissolved in assay buffer immediately before use. Increasing concentrations of protein were used with a constant, 20nM, concentration of peptide. Each well contained a final volume of 20uL in TAN buffer. Plates were incubated at 4C and data collected at 3 and 24 hours. K_d_ were estimated as the concentration of protein with half-maximal increase in fluorescence polarization.

### Biotin-streptavidin pull-down

Recombinant sE (5µg) or purified virions (2.5µg) were incubated with biotinylated DV2^419–447^ or DV2^419–447(scram)^ for 15 mins at 37°C. Streptavidin resin was incubated with 10% FBS at room temperature for 1 hr prior to use, to reduce non-specific binding. Protein and peptide mixtures were incubated with the treated resin for 30 mins at 4°C with constant rotation. Reactions were spun at 14,000rpm in a microcentrifuge at 4C for 10 mins, the supernatant aspirated, and the resin resuspended in 1 mL TAN buffer and spun again for 15 mins. After six further washes in 1 mL TAN buffer, the resin was resuspended in SDS-loading buffer, incubated for 15 mins at 100°C, and analyzed by SDS-PAGE (10 or 15%, precast gels, Biorad). Protein bands were detected by Coomassie blue staining or by immunoblotting, as indicated. Immunoblots were visualized by chemiluminescence (ECL reagents, Pierce).

### Trypsin sensitivity (content-mixing) assay

Liposomes were prepared as above, after addition of trypsin at 10 mg/ml to the lipids [33]. Unencapsulated trypsin was removed by passage of the suspension through a Superdex 200 gel filtration column (GE Healthcare). Purified virions (0.5–1 µg) were added to trypsin-containing liposomes in a final working volume of 200µL in TAN buffer. DV2 stem peptides were added at appropriate concentrations from DMSO stocks. Reactions were incubated at 37°C for 15 mins, acidified with sodium acetate pretitrated to reach a final pH of 5.5, and further incubated at 37°C for 30 mins. Reactions were neutralized with 1M TEA to a final pH of 8.0. Trypsin digestion was allowed to proceed for 1hr at 37°C. Aliquots of the reaction were resuspended in SDS-loading buffer, incubated for 20 mins at 100°C, and analyzed by SDS-PAGE followed by immunoblotting with an anti-dengue capsid antibody.

### Pyrene fluorescence fusion assay

Dengue virions containing ω-pyrene fatty acids in some of their phospholipids were produced. Briefly, BHK-21 cells were grown in the presence of 15 µg/ml of pyrene-hexadecanoic acid (Invitrogen) prior to infection with dengue virus serotype 2. Virus was harvested 24 hrs postinfection and purified as described above. Pyrene labeled virions were added to a quartz cuvette in the presence of 200 µM liposomes (prepared as described above) and brought to final volume with TAN buffer, pH 8.0. Reactions were continuously monitored with 343 nm and 470 nm as the excitation and emission wavelengths, respectively. Reactions were acidified with MES, pretitrated to yield a final pH of 5.5. Reactions were stopped by addition of 0.1% TritonX-100 to cause complete dilution of the probe. The fusion index was calculated as a percent of the loss of excimer intensity upon addition of TritonX-100.

### Cells and viruses

C6/36 cells were maintained in L-15 medium supplemented with 10% fetal bovine serum as well as penicillin, streptomycin, and ketonocazole (Invitrogen). For viral plaque assays, BHK-21 cells were seeded (5×10^4^ cells/well) in 24-well, treated tissue-culture plates in α-MEM supplemented with HEPES, pen/strep antibiotics, and 5% Fetal Bovine Serum (FBS). Cells were plated <12 hrs before use and stored at 37C with 5% CO_2_.

Dengue virus serotype 2 New Guinea Clone (NGC) was adsorbed to confluent layers of C6/36 cells for 1hr at 25°C with rocking every 15 mins. L-15 medium (Mediatech) was added, and cells were incubated at 25°C for 7 days until syncytia formation was observed. The supernatant was clarified by centrifugation at 1000 RPM for 15 mins at 4°C. Aliquots were stored at −80°C.

### Plaque assay

BHK-21 cells were seeded as described above. Aliquots from infections were thawed at 37°C in a water bath. 10 fold dilutions in Earle's balanced salt solution (EBSS) were prepared in 96-well plates by transferring 20 µl from one well to 180µl EBSS in the next. 100 µl of each dilution were added to cells. Plates were incubated for 1hr at 37°C and rocked every 15 mins. Unadsorbed virus was removed by two washes with 1 ml PBS, after which 1ml of α-MEM supplemented with carboxymethylcellulose (CMC), pen/strep antibiotics, HEPES and 2%FBS, was added to each well and incubated at 37°C for 4 days. The CMC overlay was aspirated, and cells were washed 2× with 1mL PBS and stained with crystal violet. Plates were washed with water to remove excess crystal violet and dried overnight.

### Plaque reduction inhibition assays

Cell supernatant was diluted in EBSS to a stock concentration that would allow for infection at MOI of 1, based on 50,000 seeded cells. Peptides (or carrier) were added to the inoculum as indicated for each experiment. Cells were infected for 1hr at 37°C with gentle rocking every 15 mins. Virus (or virus∶peptide mixtures) were washed from cells with 1mL of PBS (2×) and overlay medium (α-MEM supplemented with HEPES, pen/strep antibiotics and 2% FBS) added. Plates were incubated at 37°C for 24 hrs. Aliquots of the supernatant were withdrawn and stored at −80°C for plaque assay as described above.

For experiments to remove excess peptide, virus and peptide were incubated at 37°C for 15 mins and passed over a small G-50 desalting column (GE Healthcare) equilibrated with EBSS. 100 µl of the flow-through (which contained the virus∶peptide complexes) were used to infect 5×10^4^ BHK cells (MOI ∼1) in 24-well plates for 1hr at 37°C. Cells were washed 2× with PBS and overlayed with α-MEM medium supplemented with 2.0% FBS. Supernatants were harvested 24 hrs later and analyzed by the plaque assay described above.

### Virus purification

Virus was precipitated from C6/36 infected cell supernatant by addition of polyethylene glycol molecular weight 8000 (PEG8000, Sigma-Aldrich) and incubation overnight at 4°C with constant mixing. The virus was then pelleted by centrifugation for 30 mins at 10,000×g, 4°C, and the pellet was resuspended in TAN buffer and clarified by centrifugation at 5,000×g for 20 mins at 4°C. Virions were further purified on a 10% and 40% potassium tartrate with 7.5 and 30% glycerol, step gradient and centrifuged at 146,000×g for 2.5 hrs at 4°C. The gradient was harvested into 10 fractions and immunoblotted with antibodies against E (obtained from ATCC: [34]) or C (supplied by Dr. J.G. Asakov: [35]) to locate the virus. Those fractions were then pooled, concentrated and cleared of potassium tartrate and glycerol by exchange into TAN buffer using a 100K molecular weight cut-off concentrator (Amicon). Virus concentration was determined with the BCA assay (Pierce). Purified virus was stored at 4°C and used within one week of purification.

## Supporting Information

Figure S1 Binding of stem-derived peptides with DV2 sE(1–395). Binding of sE to streptavidin beads in the presence of biotinylated DV2^419–447^. Peptide was added at the concentration shown to 2.5 µg sE dimer or trimer.
(0.68 MB DOC)
