# Supplementary material for: Peptide Inhibitors of Dengue-Virus Entry Target a Late-Stage Fusion Intermediate
Source: PLoS Pathog. 2010 Apr 8;6(4):e1000851. doi: 10.1371/journal.ppat.1000851 (PMC2851732; doi:10.1371/journal.ppat.1000851)

**Supplementary Figure 1:** Binding of stem-derived peptides with DV2 sE(1-395). Binding of sE to streptavidin beads in the presence of biotinylated DV2^419-447^. Peptide was added at the concentration shown to 2.5 μg sE dimer or trimer.


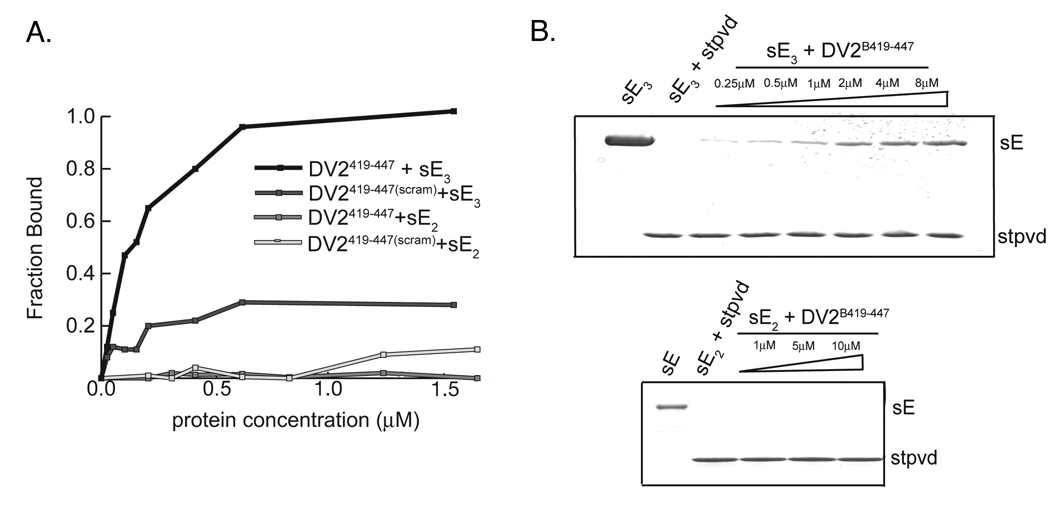

Supplement: Figure S1 — Binding of stem-derived peptides with DV2 sE(1–395). Binding of sE to streptavidin beads in the presence of biotinylated DV2419–447. Peptide was added at the concentration shown to 2.5 µg sE dimer or trimer. (0.68 MB DOC) [file ppat.1000851.s001.doc]
